# Cause and Prevention of Infant Mortality

**Published:** 1900-01

**Authors:** 


### Abstracts.

---

### CAUSE AND PREVENTION OF INFANT MORTALITY.

AT THE last meeting of the American Public Health Association, held at Minneapolis, Minn., October 31 and November 1, 2 and 3, 1899, Dr. Ernest Wende, of Buffalo, presented a report on the Cause and prevention of infant mortality.

He paid particular attention to the subject of the milk industry, which he said should be under both state and municipal control, operating in harmony under similar principles with the identical object in view, both acting together, forming a system of protection from dairy to consumer. The state should exercise its functions and control until it passes within the municipal limits and authority and should include every feature and detail in all aspects of protective sanitation. These features include briefly :

(1) The license system, with fee, periodic systematic inspections, with revoking power and penalties for dereliction; (2) the inspection system should include inspection of herds (tuberculin tests), food, animal care; (3) demand sanitary environment and buildings, correcting existing evils, and examination and approval of plans, pending erection of new ones, which should comply with a standard of sanitary construction with special reference to specific requirements of the business—namely, separate milking and stabling quarters, proper cubic air space, nonabsorbent materials; (4) scrutiny of water supply, standard feeding, health of employees, hygienic care of utensils and cans, cleanliness and protection in milking; (5) definite rules governing all procedures, particularly as to the cooling process, preparation for market, and in relation to the varying seasons; (6) the differentation of the various characters of milk by different colored or shaped cans, and being properly labelled with date of milking, time of shipment; (7) prohibition of milk preservatives and coloring agents, of adulteration, and of the marketing of milk from diseased, sick or parturient cows, of the use of disinfectants, their necessity implying bad sanitation; (8) the prohibition, particularly of refuse or swill food, upon any dairy premises of any food by reason of decomposition or fermentation considered unhealthful, and of the association of other domesticated or housing animals with cows; (9) a standard of quality, with periodic testing and analysis; (10) veterinary inspection with distinction and compensation within and quarantine against the introduction of tuberculous cattle from without the state; (11) it should be as rigid as against pestilence in man, and it is only by this means that the malady can be circumscribed and its bearing on human life favorably influenced; (12) with this standard of supervision by the state, the municipality should continue the supervision upon the same lines and which may be outlined as follows :

(*a*) Continuation of the license system with penalties, together with stringent ordinances covering quality, adulteration, sanitary care and relation to contagious disease contamination; (*b*) milk should comply with the state standard as determined by lactometer tests, periodically instituted, as ascertained both in milk houses and on wagons; (*c*) sanitary nonabsorbent milk-rooms, of determined size, air space, ventilation and light; (*d*) with specifically constructed, indirect plumbed cooling and storage boxes, and noncommunicating with sleeping rooms, privies, stables or other influencing rooms; (*e*) wagons properly lettered and numbered, with protection from summer heat; (*f*) stringent prohibition regarding intercourse with houses containing contagious diseases, particularly relating to the exchange of milk bottles and the prohibition of refilling of jars *en route;* (*g*) the constant surveillance of contagious disease in relation to milk routes by means of the "tell-tale register;" (*h*) obligatory cleansing of milk utensils by a uniform method and of cans before returning to the dairy; (*i*) special supervision of and discouragement, if not abolishment, of grocery store and similar character milk sellers, where the business is subordinate to other interests, with a consequent lack of protective facilities; (*j*) until state and city act in unison in this, where interests are identical, the adoption of arbitrary interdiction or destruction at the city line of all milk coming from dairies whose condition or herds may have a deleterious influence upon the milk; (*k*) systematic system of reports and with mutual warnings and notification of imperilling conditions.—*Boston Med. and Surg. Jour.*

